# Blind Visualization of Task-Related Networks From Visual Oddball Simultaneous EEG-fMRI Data: Spectral or Spatiospectral Model?

**DOI:** 10.3389/fneur.2021.644874

**Published:** 2021-04-26

**Authors:** René Labounek, Zhuolin Wu, David A. Bridwell, Milan Brázdil, Jiří Jan, Igor Nestrašil

**Affiliations:** ^1^Division of Clinical Behavioral Neuroscience, Department of Pediatrics, University of Minnesota, Minneapolis, MN, United States; ^2^Department of Biomedical Engineering, University of Minnesota, Minneapolis, MN, United States; ^3^Mind Research Network, Albuquerque, NM, United States; ^4^Central European Institute of Technology, Masaryk University, Brno, Czechia; ^5^Department of Biomedical Engineering, Brno University of Technology, Brno, Czechia; ^6^Center for Magnetic Resonance Research, Department of Radiology, University of Minnesota, Minneapolis, MN, United States

**Keywords:** simultaneous EEG-fMRI, task-related network visualization, spectral and spatiospectral models, visual oddball paradigm, general linear model, GLM, independent component analysis

## Abstract

Various disease conditions can alter EEG event-related responses and fMRI-BOLD signals. We hypothesized that event-related responses and their clinical alterations are imprinted in the EEG spectral domain as event-related (spatio)spectral patterns (ERSPat). We tested four EEG-fMRI fusion models utilizing EEG power spectra fluctuations (i.e., absolute spectral model - ASM; relative spectral model - RSM; absolute spatiospectral model - ASSM; and relative spatiospectral model - RSSM) for fully automated and blind visualization of task-related neural networks. Two (spatio)spectral patterns (high *δ*_4_ band and low *β*_1_ band) demonstrated significant negative linear relationship (*p*_FWE_ < 0.05) to the frequent stimulus and three patterns (two low *δ*_2_ and *δ*_3_ bands, and narrow *θ*_1_ band) demonstrated significant positive relationship (*p* < 0.05) to the target stimulus. These patterns were identified as ERSPats. EEG-fMRI F-map of each *δ*_4_ model showed strong engagement of insula, cuneus, precuneus, basal ganglia, sensory-motor, motor and dorsal part of fronto-parietal control (FPCN) networks with fast HRF peak and noticeable trough. ASM and RSSM emphasized spatial statistics, and the relative power amplified the relationship to the frequent stimulus. For the *δ*_4_ model, we detected a reduced HRF peak amplitude and a magnified HRF trough amplitude in the frontal part of the FPCN, default mode network (DMN) and in the frontal white matter. The frequent-related *β*_1_ patterns visualized less significant and distinct suprathreshold spatial associations. Each *θ*_1_ model showed strong involvement of lateralized left-sided sensory-motor and motor networks with simultaneous basal ganglia co-activations and reduced HRF peak and amplified HRF trough in the frontal part of the FPCN and DMN. The ASM *θ*_1_ model preserved target-related EEG-fMRI associations in the dorsal part of the FPCN. For *δ*_4_, *β*_1_, and *θ*_1_ bands, all models provided high local F-statistics in expected regions. The most robust EEG-fMRI associations were observed for ASM and RSSM.

## Introduction

Ives et al. and Huang-Hellinger et al. optimized initial simultaneous EEG-fMRI data acquisition (1, 2) and Allen et al. and Goldman et al. implemented first algorithms suppressing gradient MR artifacts induced in the simultaneous EEG recordings (3, 4). The development of various multimodal data fusion strategies has taken off driven by the motivation to gain the most information from EEG high temporal resolution and fMRI high spatial resolution. The first published data fusion approach cross-correlated EEG α band power fluctuations with the fMRI-BOLD signals of the resting-state paradigm (5, 6), followed by the general linear model (GLM) implementation (7, 8). The GLM became a prominent method frequently applied in the field and not only for the EEG spectra integration with resting-state (9–14) or task induced (15–20) datasets. GLMs inducing event-related potential (ERP) amplitudes or timings (21, 22), and spike-informed GLMs (23–25) have been proposed and optimized.

The voxelwise GLM results self-organize into large scale brain network (LSBN) structures (19). Concurrent fusion strategy often rotates fMRI data into space of linearly mixtured spatially independent components, i.e., the LSBNs, with their representative clusterwise induced BOLD fluctuations, which are compared to various EEG dynamics (26–31). In parallel, regression or correlation approaches inferring EEG and fMRI dynamics, joint independent component analysis (32), graph build approach (33), dynamic functional connectivity (34), or multimodal dynamic causal modeling (35) have been proposed to fuse EEG-fMRI data with various result interpretations. Many regression or deconvolution approaches reported that EEG-fMRI hemodynamic response function (HRF) demonstrates varying timings and shapes (10, 19, 20, 36–42). In physiologic situations, the BOLD signal is delayed to EEG events but an extreme example is the epileptic spike EEG-fMRI where BOLD signal peaks can precede the EEG spikes (41, 42). Thus, it is a preferable approach to model variable HRF than to use fixed canonical HRF, which has still been dominating in the common practice (5–9, 11–18, 21–26, 28–33, 43).

Over various existing EEG-fMRI data fusion techniques, the ability to blindly and automatically visualize and quantify robust task-related functional networks and their EEG-fMRI associations (e.g., *via* variable HRF) is lacking. We have focused on the simple GLM fusion approach with variable HRF aggregating automatically induced EEG spectra (19, 20) and tested whether we can identify fusion settings that blindly visualizes task-related networks. This automated method may offer high reproducibility with tremendous potential in the clinical research or even clinical practice applications to quantitatively measure cognitive dysfunction.

Cognitive dysfunction may occur in various neurologic and psychiatric conditions including epilepsy and can be estimated from EEG, e.g., by measuring cognitive event-related responses such as P300 potential. The P300 response is time-locked to an event and is elicited by a task/event when a tested individual is requested to respond to a single stimulus or a set of stimuli as in the oddball paradigm. The P300 has been increasingly investigated as a marker of cognitive processing. Specifically, the P300 response represents a neural signature of the processing of stimulus context depending on the attention and state of arousal leading to an appropriate response (44). Although the P300 has been almost exclusively assessed in the temporal domain *via* ERPs (45, 46), the characterization in the frequency/spectral domain, since time and frequency are fully complementary domains, may provide additional insight into the data. Spectral (Equation 1) (16, 17, 43, 47) and spatiospectral (Equation 2) (19, 43) models have already been proposed for the blind visualization of task-related networks from simultaneous EEG-fMRI data. The 1^st^ model (Equation 1) assumes that local fMRI BOLD signal fluctuations (b) are proportional to fluctuations of the frequency-specific (*ω*) weighted EEG absolute/relative power spectra (*p*) with modeled between-signal delay *via* HRF convolution kernel (*h*). The weighting function *g*(*ω*) can be considered a frequency specific filter modulating the power spectra and final power fluctuations are often estimated as an average over channels (16, 17, 43, 47). The 2^nd^ model (Equation 2) is similar but considers the filtering property to be channel (*c*) specific. The identification of a robust task-related weighting function *g(ω)* or *g(c,ω)*, i.e., event-related (spatio)spectral patterns (ERSPat) in EEG spectra, remains a not fully optimized challenge in the fusion process.

(1) b∝(∫g(ω)p(ω) dω)∗h

(2) b∝(∫∫g(c,ω)p(c,ω) dc dω)∗h

The EEG absolute/relative power spectra consist of a linear mixture of *stable* spatiospectral patterns [i.e., different stable *g(c,ω)* functions in Equation (2)] with temporal fluctuations that were more task-related for the relative power rather than the absolute power (48, 49). Absolute EEG power identified 14 stable patterns with highly significant EEG-fMRI associations at visual oddball dataset (19, 48). Relative EEG power identified 10 stable patterns similar to the absolute power patterns and two other relative power specific stable patterns, for which the EEG-fMRI relationships have not been investigated yet (49).

Several studies utilized the spectral model for the visualization of task-related neuronal networks from EEG-fMRI data (15–18) or the spatiospectral model (19, 43) with few mutual discrepancies: (i) the response function was fixed or variable; (ii) different tasks were used. Therefore, a direct and fair comparison between models still needs to be investigated.

Within the current study, we are presenting the full comparison between absolute/relative power based spectral (Equation 1) and spatiospectral (Equation 2) models for fully automatic EEG-fMRI fusion. The goal is to optimize the automatic visualization and quantification of task-related neuronal networks. The robustness over models was objectively assessed.

## Materials and Methods

### Experimental Design

The identical simultaneous EEG-fMRI dataset of visual oddball paradigm was used as previously described (18, 19, 48, 49). The event-related designed visual oddball task was performed by 21 healthy subjects (13 right-handed men, one left-handed man, seven right-handed women; age 23 ± 2 years). Three stimulus types were presented randomly to each subject. Each stimulus consisted of a single yellow uppercase letter shown for 500 ms on a black background. Inter-stimulus intervals were either 4, 5, or 6 s (drawn uniformly and randomly). A total of 336 stimuli (divided into four consequential sessions) were presented, consisting of targets (letter X, 15%), frequents (letter O, 70%), and distractors (letters other than X and O, 15%). Subjects were instructed to press a button on the box held in their right hand whenever the target stimulus appeared and not to respond to distractor or frequent stimuli.

This study was approved by and carried out in accordance with the recommendations of the Masaryk University Ethics committee guidelines and all subjects signed the approved written informed consent in accordance with the Declaration of Helsinki.

### Simultaneous EEG-fMRI Data Acquisition

The scalp EEG data, with reference between Cz and Fz electrodes, were acquired with an MR compatible 32-channel 10/20 EEG system (*BrainProducts, Germany*) and a sampling frequency of 5 kHz. Two channels were used for ECG and EOG. *Via* the BrainVision Recorder system (*BrainProducts, Germany*), the EEG data were synchronized and acquired simultaneously with fMRI data during gradient echo imaging sequences [1.5 T Siemens Symphony scanner equipped with Numaris 4 System (*Mrease*)]. Gradient echo, echo-planar functional imaging sequence was acquired with following parameter setting: TR = 1,660 ms; TE = 45 ms; FOV = 250 × 250 mm; FA = 80°; matrix size = 64 × 64 (3.9 × 3.9 mm); slice thickness = 6 mm; 15 transverse slices covering the whole brain except the inferior part of the cerebellum. The task was divided into four equal runs of 256 scans and 84 stimuli.

Following simultaneous EEG-fMRI acquisition, high-resolution anatomical T1-weighted images were acquired using an MPRAGE sequence with 160 sagittal slices, matrix size 256 × 256 resampled to 512 × 512; TR = 1,700 ms; TE = 3.96 ms; FOV = 246 mm; FA = 15°; and slice thickness = 1.17 mm.

### EEG Data Preprocessing

The EEG data were preprocessed as described in (19, 48) using BrainVision Analyzer 2.02 (*BrainProducts, Germany*) with the implemented manufacturer's pipeline. Gradient artifacts were removed using average artifact subtraction (used sliding window with window length=21^*^TR) at the acquisition sampling rate 5 kHz (3) and filtered with a Butterworth zero phase 1–40 Hz band-pass filter. Then, EEG signals were resampled to 250 Hz (antialiasing filter included). Ballistocardiogram (BCG) artifacts were removed by average artifact subtraction (used sliding window with window length = 21^*^BCG epochs) waveform from each channel (50) and signals were re-referenced to the average. Eye-blinking artifacts were removed by conducting a temporal ICA decomposition and removing eye-blink artifacts from the back-reconstructed time course.

### EEG Spatiospectral Decomposition

The decomposition was the same as implemented and previously described (48, 49). The preprocessed EEG signal from each lead and session was normalized to 0 mean and variance 1, and divided into 1.66 s (repetition time of fMRI scanning TR) epochs without overlap. Each epoch was transformed to a spectral domain with the fast Fourier transform (FFT), generating a vector (length = 67) of complex valued spectral coefficients between 0 and 40 Hz. Complex values were converted to absolute power by taking the absolute value and squaring, or converted to relative power value by dividing the squared value by the power of the whole epoch. The output vector of 67 real absolute/relative power values comprised a 3D matrix ***E*** with dimensions *n*_*t*_, *n*_*c*_, and *n*_*ω*_. The dimension *n*_*t*_ represents the number of spatiospectral epochs (*n*_*t*_ = 256), the dimension *n*_*c*_ is the total number of leads (*n*_*c*_ = 30), the dimension *n*_*ω*_ is the total number of spectral coefficients (*n*_*ω*_ = 67). The EEG spatiospectral decomposition (Equation 3) decomposes the matrix of spatiospectral maps ***E*** into a source matrix ***S*** of dimensions ***S****(*${m,n}_{c}^{*}$n_*ω*_*)* containing independent spatiospectral patterns and a mixing matrix ***W*** of dimensions ***W****(n*_*t*_*,m)* containing the patterns' dynamics. Dimension *m* is the number of decomposed independent spatiospectral components (*m* = 20).

(3) $$\begin{array}{l} \text{E}=\text{W}\text{S} \end{array}$$

Using the GIFT toolbox (http://mialab.mrn.org/software/gift/) (51), the matrix ***E*** was dimensionally reduced using PCA (single-subject reduction to 50 principle components and group-based reduction to 20 components), followed by INFOMAX group-ICA (52) with 10 ICASSO runs (53).

Only spatiospectral patterns, which were reported to be stable and observed in both absolute/relative power spectra (48, 49), i.e., 10 patterns, have been selected from output source matrices ***S*** of separate group-ICA runs for absolute/relative powers.

Individual subject's time courses were generated by PCA based back-reconstruction (i.e., the individual partition of the PCA reducing matrix is matrix multiplied by the individual partition of the aggregate reducing matrix) (51) of the group spatiospectral patterns on the individual subject spatiospectral maps and time-courses.

### Selection of Stable EEG Spatiospectral Patterns With Relationship to the Task

For each subject and session, we have one matrix ***W*** with dimensions ***W*** *(n*_*t*_, *20)* containing the back-reconstructed time course of each spatiospectral component. Let a matrix ***U*** of dimensions ***U*** (4^*^n_*t*_,10) to be a single-subject matrix of fluctuations of stable spatiospectral patterns over all four sessions. Relationships between these dynamics and stimulus vector timings (in matrix ***X***) were assessed with a single-subject general linear model (Equation 4, GLM) (54) solved with the least mean square algorithm (Equation 5) and a continuous group one-sample *t*-test for the *k-th* stimulus vector (Equation 6) (48). Variable *c* is the vector of binary positive contrast at the stimulus vector of interest, the brackets < > characterize the expectation over subjects, variable σ is the standard deviation and variable *s* is the total number of subjects. Model matrix ***X*** contained frequent, target and distractor timings in 12 separate binary vectors for each stimulus and session and four vectors for the DC component in each session.

(4) U=Xβ+ϵ

(5) $$\begin{array}{l} \beta ={\left({\text{X}}^{T}\text{X}\right)}^{-1}{\text{X}}^{T}\text{U} \end{array}$$

(6) $$\begin{array}{l} t_{k}=\sqrt{s}\frac{\langle {\text{c}}_{k}^{T}\beta_{k}\rangle }{\sigma_{\langle {\text{c}}_{k}^{T}\beta_{k}\rangle }} \end{array}$$

These spatiospectral patterns, where any |t|-value was higher than 3.25 (critical value at *p*_FWE_ < 0.05 for 10 multiple comparisons, i.e., 10 selected stable patterns, and 16 degrees of freedom, i.e., 16 variables in model matrix ***X***) for any stimulus type in absolute or relative power, were considered as a pattern with task-related EEG power fluctuations. Second selection criteria was to demonstrate mean |t|-value averaged over all spatiospectral/spectral models with *p* < 0.05 (~|t| > 2.0) with relatively small standard deviation in the averaged |t|-value over models, i.e., STD_|t|_ < 0.5.

### Task-Related EEG Spectral Patterns

All 10 stable spatiospectral patterns observed in both power types were averaged over leads and provided 10 spectral filters *g(ω)* (Equation 1). The filters were used for filtering of power spectra of matrix ***E*** reshaped at dimensions ***E***(*n*_*t*_, *n*_*c*_, *n*_*ω*_). Separate for each filter, time point and channel, the absolute/relative power value *p(t,c)* was filtered as Equation 7. Time-course *p(t)* of each channel *c* was normalized to mean 0 and variance 1, and final absolute/relative power fluctuation $\bar{p\left(t\right)}$ was estimated as an average over channels for each specific spectral pattern.

(7) $$\begin{array}{l} p\left(t,c\right)=\overset{n_{\omega }}{\underset{\omega =1}{\sum }}g\left(\omega \right)E\left(t,c,\omega \right) \end{array}$$

Task-related spectral patterns were evaluated and identified with the same methodology as described in sub-chapter Selection of Stable EEG Spatiospectral Patterns With Relationship to the Task, but matrix ***U*** (Equations 4, 5) consisted of 10 pattern-specific averaged $\bar{p\left(t\right)}$ fluctuations.

### fMRI Data Preprocessing

The fMRI data were preprocessed with SPM8 (*Wellcome Trust Center for Neuroimaging, London, UK*) software library. Motion artifacts were minimized by alignment of all functional scans, followed by co-registration with the subject's anatomical image and normalization into standardized MNI space (*Montreal Neurological Institute* template) (55). Functional scans were spatially smoothed with an isotropic 3D Gaussian filter (FWHM = 8 mm) to increase the signal to noise ratio (SNR) and to make the random errors more normally distributed. Periods longer than 128 s were linearly detrended to remove slow drifts and physiological noise.

### EEG-fMRI General Linear Modeling With Variable HRFs

Relationships between fMRI voxel time-courses (***Y***) and EEG task-related spectral/spatiospectral pattern time-courses were examined using the individual GLMs (Equation 8) (54) with the EEG time course convolved with the canonical HRF (***x***_1_), convolved with the 1^st^ temporal derivative of the HRF (***x***_2_) or convolved with the 2^nd^ temporal derivative of the HRF (***x***_3_) (19, 20). In addition to the three EEG regressors ***x***_1_*-****x***_3_, the model matrix ***X*** contained a DC component. Regression matrices ***β*** were estimated over all GLMs with the ReML algorithm (Restricted Maximum Likelihood) implemented in SPM12 software (*Wellcome Trust Center for Neuroimaging, London, UK*) in the MATLAB programing environment (*MathWorks, Natick, USA*).

(8) Y=Xβ+ϵ

Group-averaged EEG-fMRI results were estimated with a one-way ANOVA test (implemented in SPM12) of three EEG-derived single-subject spatial β-maps for each of three EEG regressors. The β weights served as dependent variables in separate ANOVA tests conducted for each spectral/spatiospectral pattern, generating group-averaged spatial EEG-fMRI F-maps. The final F-maps were thresholded for objective evaluations at *p* < 0.001 uncorrected for multiple statistical tests (i.e., with a critical absolute *F*-value of 5.7), and the criteria that clusters contain 100 voxels or more. For visualizations, the F-maps were thresholded at *p* < 0.0001 (i.e., F > 8.1) due to high result robustness.

Group-averaged EEG-fMRI HRFs (***h***_*i*_) in each voxel *i* were estimated from group-averaged regression coefficients ***β*** (estimated within Equation 8) with Equation 9 where ***r*** is the canonical HRF and numbers 1–3 are indexes of regressors ***x***_1_*-****x***_3_ (19, 56).

(9) $$\begin{array}{l} {\text{h}}_{i}=\beta_{i,1}\text{r}+\beta_{i,2}\frac{d\text{r}}{dt}+\beta_{i,3}\frac{d^{2}\text{r}}{dt^{2}} \end{array}$$

### Assessment of EEG-fMRI Results

Group-averaged EEG-fMRI F-maps and HRFs were visually inspected for a subjective similarity/dissimilarity evaluation over the similar spectral/spatiospectral patterns. For the objective assessment, volume, mean *F*-value, median *F*-value, and maximal *F*-value were automatically extracted from the suprathreshold voxels (*p* < 0.001) of the final group-averaged EEG-fMRI F-maps of every stable task-related spectral/spatiospectral pattern. The spectral/spatiospectral model with the highest objective metrics was considered as the most successful one in blind visualization of a task-related neural network.

## Results

### Task-Related EEG Spectral/Spatiospectral Patterns

**Figure 1:**
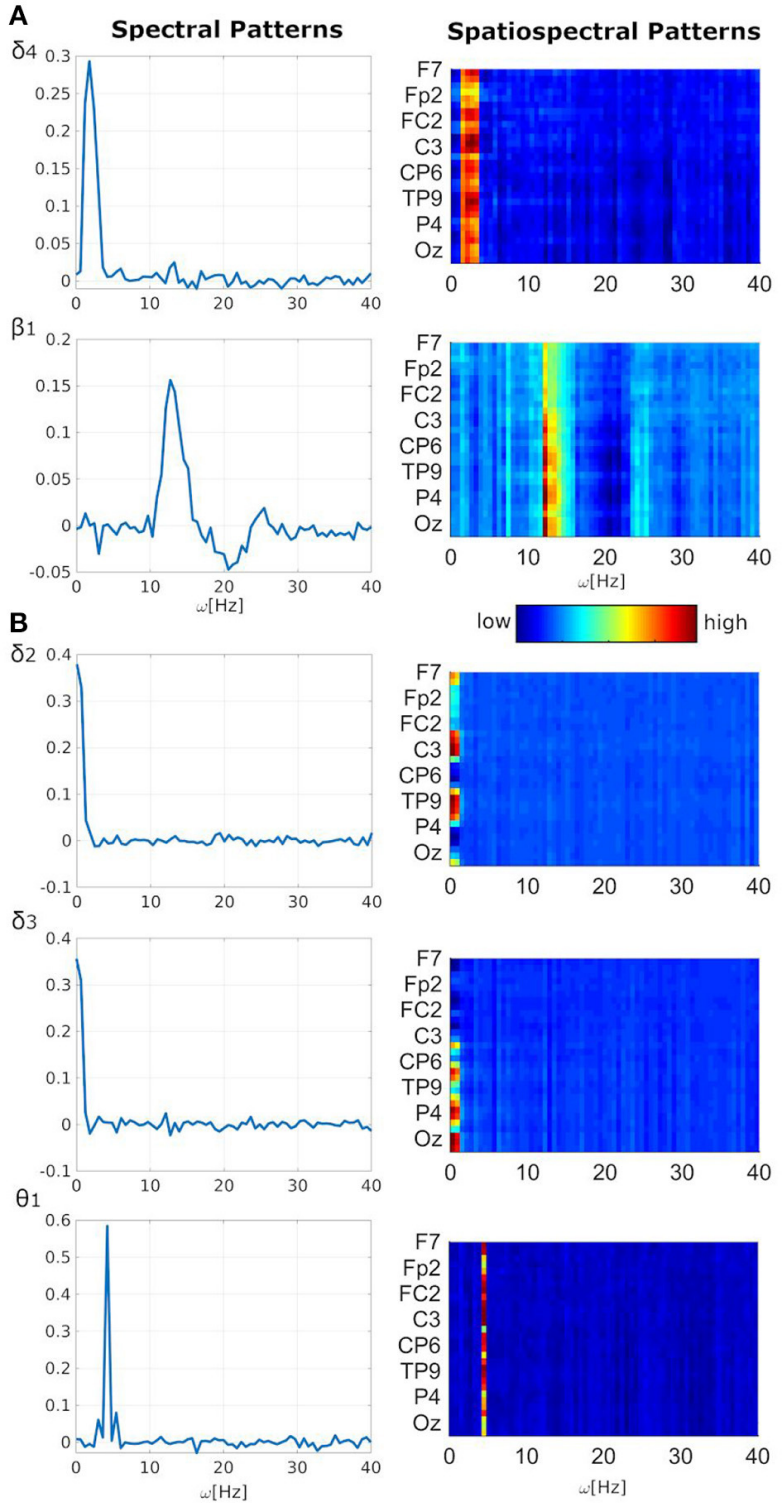
Task-related EEG spectral or spatiospectral patterns. **(A)** Frequent related; **(B)** target related. Pattern shortcuts and indexes (e.g., δ_4_) are the same as used in (19) for consistency purposes. All spectral patterns are averages over electrodes of both absolute or relative spatiospectral patterns. Patterns' amplitudes (i.e., y-axis for spectral patterns and color-coding for spatiospectral patterns) are in arbitrary units. For spatiospectral patterns, the dark blue color is an approximate minimum value in the y-axis of the corresponding spectral pattern. The dark red color is an approximate maximum value in the y-axis of the corresponding spectral pattern.

Significant negative linear relationship between EEG fluctuations and frequent stimulus was observed in two spectral/spatiospectral patterns (i.e., δ_4_ with inter-model *t*-value −3.64 ± 1.00, β_1_ with *t*-value −3.35 ± 0.17, Table 1, Figure 1A, *p*_FWE_ < 0.05). The negative linear relationship can be interpreted as the EEG pattern power decrease during the frequent stimulus onset. Other three patterns (i.e., δ_2_ with *t*-value 2.46 ± 0.43, δ_3_ with *t*-value 2.34 ± 0.11, θ_1_ with *t*-value 2.05 ± 0.33) demonstrated a significant positive linear relationship between the EEG power fluctuations and target stimulus (Table 1, Figure 1B, *p* < 0.05 and *STD*_|t|_ < 0.5). The positive linear relationship can be interpreted as the EEG pattern power increase during the target stimulus onset. The δ_4_ pattern fluctuations with *t*-value −2.39 ± 0.62 may also be sensitive to distractor stimulus with similar negative linear relationship (Table 1, Figure 1A, *p* < 0.05) as observed for the frequent stimulus. Lower robustness of relationships between EEG patterns and target or distractor stimuli might be caused by lower stimulus amounts. Five of 10 analyzed stable EEG patterns demonstrated potentially significant relationship to stimuli vectors of the visual oddball task (Table 1, Figure 1).

**Table 1:** Group *t*-values of linear relationship between stable EEG pattern fluctuations and stimuli vectors.

		**Spectral**		**Spatiospectral**		**Mean**	**STD**
		**Absolute**	**Relative**	**Absolute**	**Relative**		
Frequent	δ_1_	−0.09	0.46	−1.13	−0.58	−0.34	0.59
	δ_2_	**−5.24**	−2.42	−0.81	−0.92	−2.35	1.79
	δ_3_	**−3.71**	−1.71	−1.45	−1.45	−2.08	0.95
	δ_4_	**−3.44**	**−4.70**	−2.09	**−4.32**	**−3.64**	1.00
	θ_1_	−1.76	−0.87	−0.34	−2.03	−1.25	0.68
	θ_4_	−2.54	−1.95	0.39	−1.01	−1.28	1.10
	α_1_	1.65	**3.54**	1.72	2.59	2.38	0.77
	α_3_	0.62	2.03	2.48	0.12	1.31	0.97
	β_1_	**−3.64**	**−3.25**	−3.23	**−3.28**	**−3.35**	0.17
	β_2_	−1.89	−0.64	−0.17	**−3.74**	−1.61	1.38
Target	δ_1_	2.80	**3.35**	−0.40	−1.86	0.97	2.18
	δ_2_	2.53	2.93	1.77	2.63	2.46	0.43
	δ_3_	2.26	2.20	2.43	2.46	2.34	0.11
	δ_4_	1.08	0.35	0.24	1.04	0.68	0.39
	θ_1_	2.18	1.49	2.37	2.17	2.05	0.33
	θ_4_	1.93	0.65	0.39	1.45	1.11	0.62
	α_1_	2.05	0.51	2.62	1.73	1.73	0.77
	α_3_	0.93	0.17	0.58	0.21	0.47	0.31
	β_1_	−0.11	−2.30	−0.52	−1.44	−1.09	0.85
	β_2_	−0.82	−2.06	−0.06	−1.40	−1.08	0.74
Distractor	δ_1_	−0.19	0.30	0.56	0.21	0.22	0.27
	δ_2_	−1.30	−0.40	0.31	1.30	−0.02	0.95
	δ_3_	−1.05	−0.56	0.21	−0.35	−0.44	0.45
	δ_4_	−3.11	−2.76	−2.24	−1.46	−2.39	0.62
	θ_1_	−0.58	1.45	−0.25	0.01	0.16	0.77
	θ_4_	−2.06	−2.52	−1.00	−0.43	−1.50	0.83
	α_1_	−0.23	1.17	0.56	1.65	0.79	0.70
	α_3_	−0.39	0.60	−0.34	0.40	0.07	0.44
	β_1_	−0.79	0.19	−0.61	−0.97	−0.55	0.44
	β_2_	−1.06	−1.00	0.12	−1.31	−0.81	0.55

*Bold highlighted t-values demonstrated significant relationship with p_FWE_ < 0.05. Green-color highlighted rows indicate the patterns with significant task-related relationships. These patterns demonstrated p_FWE_ < 0.05 or a mean over models of p < 0.05 (i.e., mean |t| > 2.0) with t-value standard deviation of STD_|t|_ < 0.5*.

### Frequent-Related EEG-fMRI Networks

**Figure 2:**
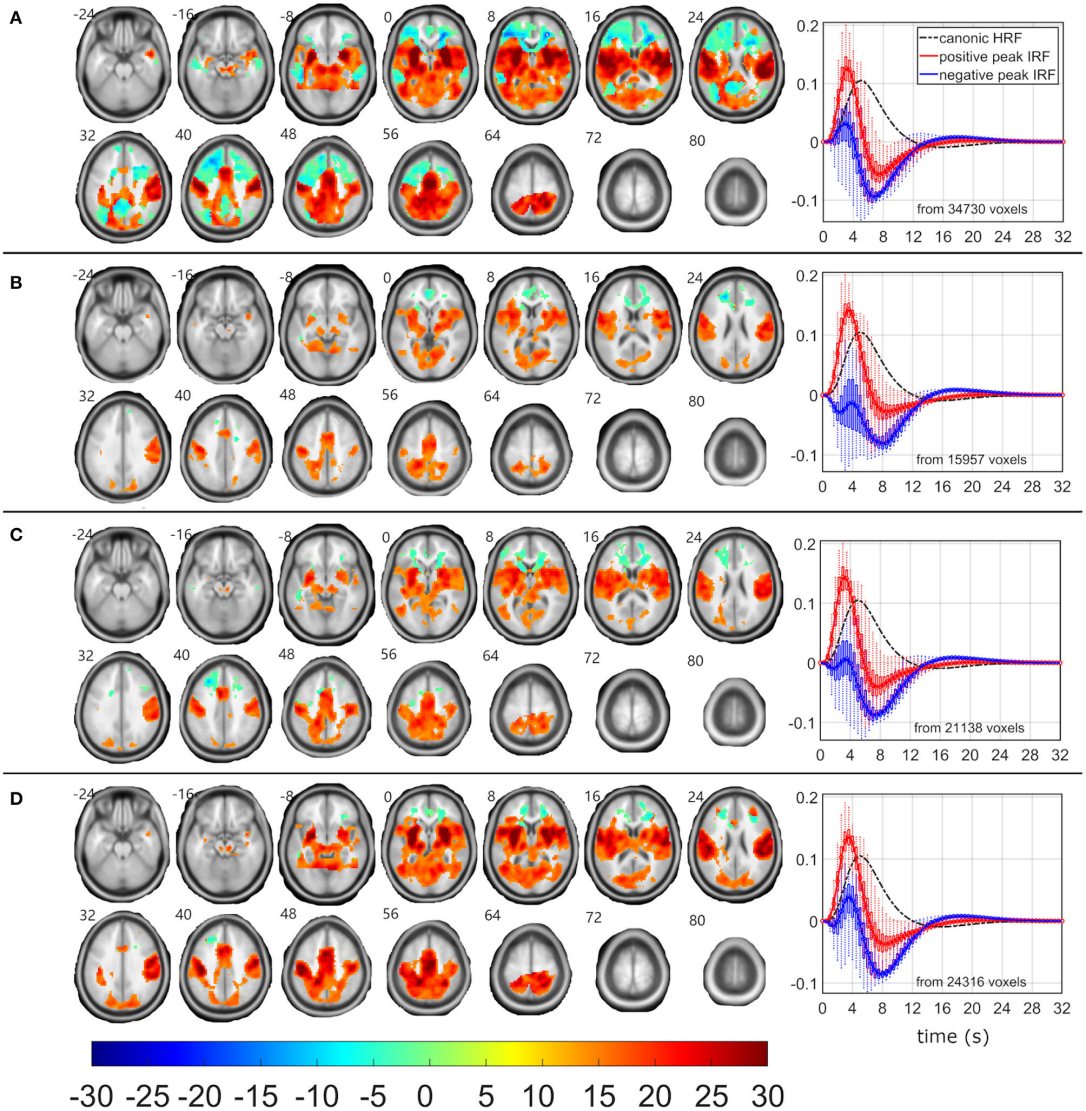
EEG-fMRI F-maps and estimated hemodynamic response functions for all investigated models of the δ_4_ pattern. The EEG-fMRI F-map colorbar is the same over all models. HRF, hemodynamic response function; IRF, impulse response function. If HRF demonstrated a reduced peak and an amplified trough (i.e., blue color-coded HRFs) the *F*-values were assigned with a negative sign. The F-maps were threholded with *p* < 0.0001, i.e., |F| > 8.1. The voxels with |F| > 12.1 met the condition *p*_FWE_ < 0.05. The red color coded HRFs were derived from voxels with positive signed suprathreshold *F*-values. All F-maps are shown following the neurological convention, i.e., left hemisphere on the left side of the axial slice. **(A)** Absolute spectral. **(B)** Relative spectral. **(C)** Absolute spatiospectral. **(D)** Relative spatiospectral.

As the δ_4_ pattern demonstrated the highest level of relationship between pattern fluctuations and frequent stimulus, the EEG-fMRI F-maps also visualized the highest *F*-values and largest amounts of supra-threshold voxels over all four investigated EEG patterns (Table 2, Figure 2). The absolute spectral model (ASM) provided the largest and the most significant EEG-fMRI associations in comparison to other models (Table 2). Still, statistical measures are very high and robust in all investigated models for the δ_4_ pattern (Table 2).

**Table 2:** Spatial statistics of task-related EEG-fMRI F-maps.

			**Spectral**		**Spatiospectral**	
			**Absolute**	**Relative**	**Absolute**	**Relative**
Frequent related	δ_4_	Volume [mm^3^]	**937,845**	431,865	571,266	656,910
		Mean *F*-value	**14.08**	9.96	10.65	13.05
		Median *F*-Value	**12.48**	9.04	9.57	11.4
		Max *F*-value	**53.83**	27.35	32.19	45.27
	β_1_	Volume [mm^3^]	**282,555**	-	40,797	106,704
		Mean *F*-value	**8.37**	-	7.4	7.61
		Median *F*-Value	**7.76**	-	7.19	7.23
		Max *F*-value	**22.39**	-	12.17	15.31
Target related	δ_2_	Volume [mm^3^]	**524,205**	26,271	58,185	26,865
		Mean *F*-value	**10.14**	7.34	7.68	7.35
		Median *F*-Value	**9.14**	7.00	7.26	6.96
		Max *F*-value	**28.86**	13.76	16.12	14.97
	δ_3_	Volume [mm^3^]	**658,557**	15,525	2,646	17,118
		Mean *F*-value	**10.64**	7.54	7.01	7.45
		Median *F*-Value	**9.45**	7.05	6.81	6.98
		Max *F*-value	**36.09**	16.5	9.57	14.03
	θ_1_	Volume [mm^3^]	**852,930**	140,994	236,844	334,746
		Mean *F*-value	**12.44**	7.94	8.92	9.02
		Median *F*-Value	**11.08**	7.41	8.25	8.19
		Max *F*-value	**47.32**	17.1	21.24	29.78

*The values were estimated from all supra-thresholded voxels with p < 0.001 (i.e., |F| > 5.7). Bold highlighted numbers are the highest obtained values over investigated models per metric. Values |F| > 8.1 met the condition p < 0.0001, values |F| > 12.1 met the condition p_FWE_ <0.05*.

Positive δ_4_ pattern demonstrated significant (*p*_FWE_ < 0.05) bilateral EEG-fMRI associations in cuneus, precuneus, insula, basal ganglia, and in sensory-motor network, somatosensory network and dorsal parts of the fonto-parietal control network (FPCN) (57–60) (Figure 2). Putamen, pallidum, thalamus, and brainstem were involved within subcortical gray matter structures (Figure 2). The dorsal parts of the FPCN overlap with the dorsal attention network (DAN) (57) and may be no discernable one from the other in a lower spatial resolution (Figure 2). Spatial distribution in Figure 2B represents the same result, which was presented in (19) with absolute spatiospectral model (ASSM) and variable HRF modeling. Here, we proposed that the ASM with variable HRF modeling or relative spatiospectral model (RSSM) with variable HRF modeling increased the statistical power and the robustness (Table 2, Figure 2). All models demonstrated a positive HRF peak faster than a peak timing of widely used canonical HRF (Figure 2) and noticeable HRF trough (Figure 2) in the insula, sensory-motor network, somatosensory network, dorsal part of the FPCN and basal ganglia. Except this expected response (i.e., red HRFs in Figure 2), every model detected brain areas with reduced HRF peak followed by larger HRF trough amplitude (i.e., the blue HRFs in Figure 2). Trough peaks in both detected HRFs were again faster than an expected trough timing for the canonical HRF (Figure 2). The HRF with a reduced peak and an amplified trough was observed in areas of superior frontal cortex and parietal cortex (Figure 2A), which might belong to the frontal parts of the FPCN (57–60) or default mode network (DMN) (57). Inferiorly, we noticed significant (*p*_FWE_ < 0.05) bilateral cluster spots in frontal white matter areas (Figure 2A) where forceps minor, anterior thalamic radiation and inferior fronto-occipital fasciculus might pass [evaluated by a visual inspection of EEG-fMRI F-maps overlaid with the JHU white-matter atlas (61–63) in the MNI space].

**Figure 3:**
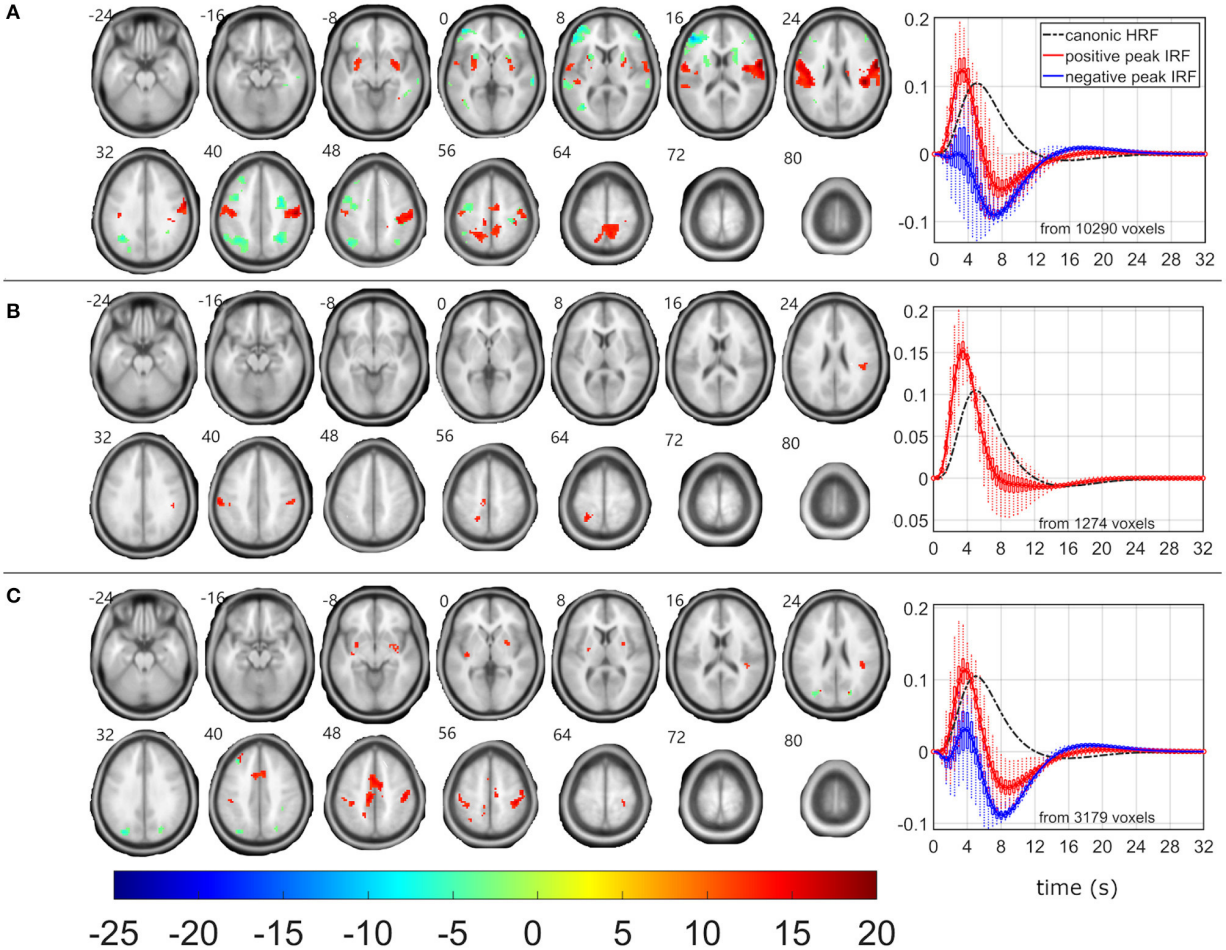
EEG-fMRI F-maps and estimated hemodynamic response functions for all investigated models of the β_1_ pattern. The EEG-fMRI F-map colorbar is the same over all models. HRF, hemodynamic response function; IRF, impulse response function. If HRF demonstrated a reduced peak and an amplified trough (i.e., blue color-coded HRFs) the *F*-values were assigned with a negative sign. The F-maps were threholded with *p* < 0.0001, i.e., |F| > 8.1. The voxels with |F| > 12.1 met the condition *p*_FWE_ < 0.05. The red color coded HRFs were derived from voxels with positive signed suprathreshold *F*-values. All F-maps are shown following the neurological convention, i.e., left hemisphere on the left side of the axial slice. **(A)** Absolute spectral. **(B)** Relative spectral. **(C)** Absolute spatiospectral. **(D)** Relative spatiospectral.

As the β_1_ pattern demonstrated similar negative linear relationship between its power fluctuation and the frequent stimulus (Table 1), the EEG-fMRI F-maps demonstrated similar locations of association spots (Figure 3) where maximal |F| values were observed in the δ_4_ EEG-fMRI F-maps (Figure 2), and similar HRF properties (Figure 3). The β_1_ EEG-fMRI F-maps demonstrated a lower statistical robustness for all models (Figure 3, Table 2) when compared to the δ_4_ F-map robustness (Figure 2, Table 2). Again, the ASM provided the most robust statistics at the inter-model comparison level (Table 2). The relative spectral model (RSM) did not demonstrate any significant EEG-fMRI associations, which is analogical to the previous observation of no relative β associations with the fixed canonical HRF at the same dataset (18). Again, the RSSM was more robust than the ASSM (Table 2). Overall, the lower robustness, the β_1_ EEG-fMRI associations might appear more spatially specific. As interpreted from the ASM EEG-fMRI β_1_ F-map (Figure 3). From the basal ganglia, the bilateral putamen demonstrated EEG-fMRI associations with normal HRF peak. From the sensory-motor network, bilateral EEG-fMRI associations with normal HRF peak were observed in primary sensoric, premotor, somatosensory cortices, supramarginal gyrus, left lateralized Brodmann area 7 (BA7), and premotor BA6. The reduced HRF peak properties were observed for lateralized BA9, BA10, BA46, and right hand primary motor cortex. The RSSM emphasized somatosensory BA6 associations compared to the ASM results (Figure 3). In regards to the mentioned spatial specificity, it is important to note that one can get similar spatial distribution for the δ_4_ EEG-fMRI F-map if stricter threshold than *p* < 0.0001 is set in the Figure 2 visualization where local spatial statistics exceeded the β_1_ pattern results (Table 2).

### Target-Related EEG-fMRI Neworks

**Figure 4:**
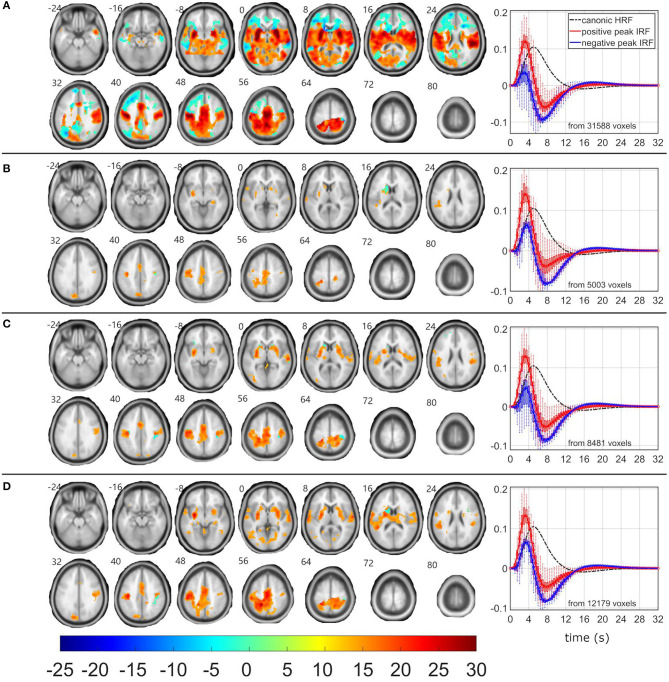
EEG-fMRI F-maps and estimated hemodynamic response functions for all investigated models of the θ_1_ pattern. The EEG-fMRI F-map colorbar is the same over all models. HRF, hemodynamic response function; IRF, impulse response function. If HRF demonstrated a reduced peak and an amplified trough (i.e., blue color-coded HRFs) the *F*-values were assigned with a negative sign. The F-maps were thresholded with *p* < 0.0001, i.e., |F| > 8.1. The voxels with |F| > 12.1 met the condition *p*_FWE_ < 0.05. The red color coded HRFs were derived from voxels with positive signed suprathreshold *F*-values. All F-maps are shown following the neurological convention, i.e., left hemisphere on the left side of the axial slice. **(A)** Absolute spectral. **(B)** Relative spectral. **(C)** Absolute spatiospectral. **(D)** Relative spatiospectral.

Although δ_2_, δ_3_, and θ_1_ patterns demonstrated all the positive relationship (*p* < 0.05 and STD_|t|_ < 0.5) between EEG pattern fluctuations and target stimulus, the EEG-fMRI data fusion visualized the largest and most robust F-maps for the θ_1_ pattern over all investigated models (Table 2, Figure 4). The most robust statistics was yielded by ASM, followed by RSSM, ASSM, and RSM (Table 2). All models emphasized left lateralized EEG-fMRI associations in the sensory-motor network (corresponding to the push on the right hand held button box and the target push button response) with smaller amounts of the basal ganglia associations, which were somewhat preserved for the ASM and partly for the RSSM (Figure 4). The ASM preserved significant EEG-fMRI associations in the dorsal parts of the FPCN overlapping with DAN (Figure 4). These EEG-fMRI associations presented a non-reduced HRF peak and a noticeable HRF trough again with timing faster than classic canonical HRF (Figure 4). The ASM still visualized a significant (*p*_FWE_ < 0.05) reduced HRF peak and an amplified HRF trough in areas of the frontal parts of the FPCN, DMN and superior frontal white matter but in smaller amounts than observed for the frequent-related δ_4_ pattern (Figures 2, 4). The RSM, ASSM, and RSSM revealed a comparably smaller amount of significant clusters in locations as ASM (Figure 4).

**Figure 5:**
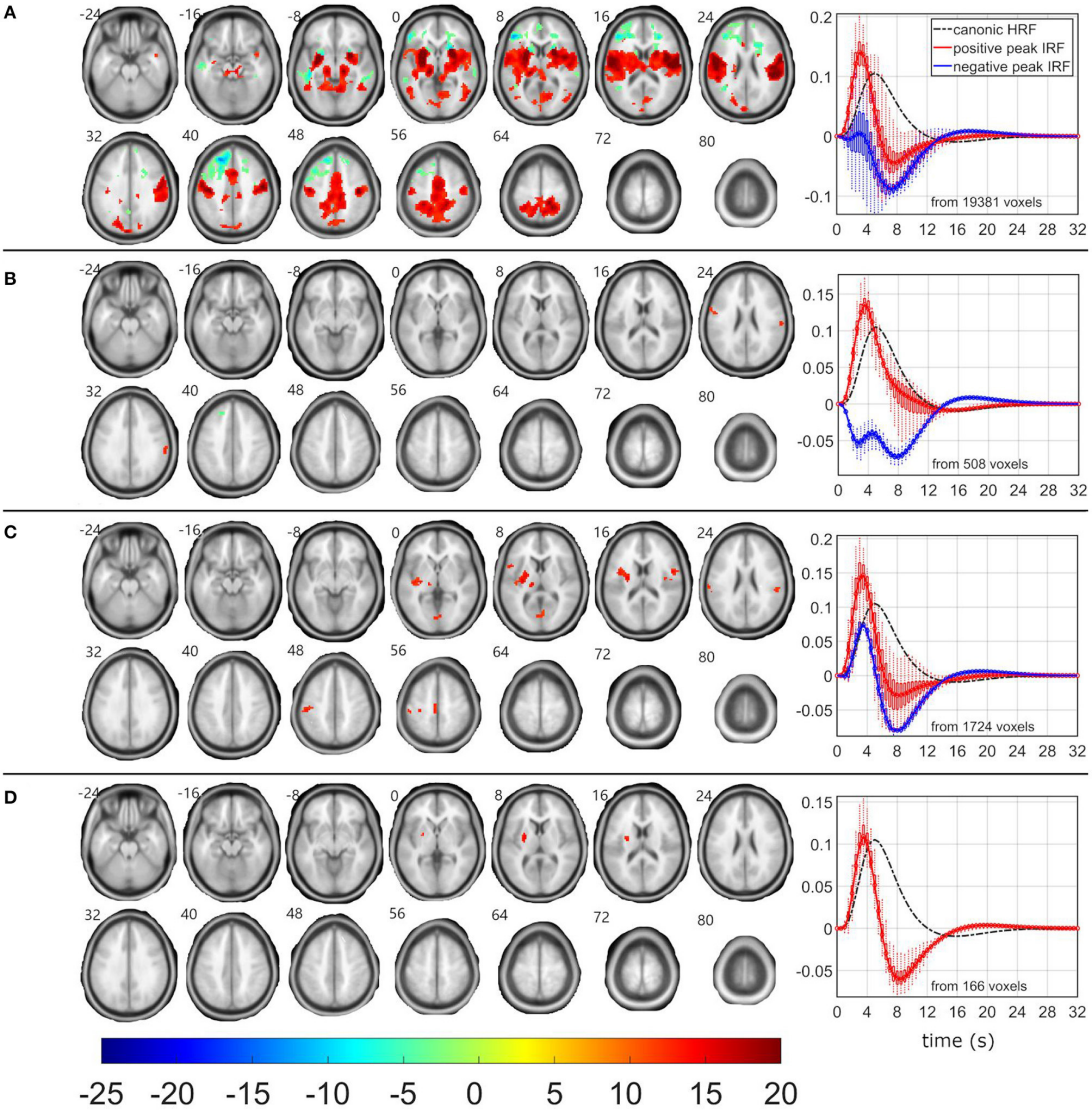
EEG-fMRI F-maps and estimated hemodynamic response functions for all investigated models of the δ_2_ pattern. The EEG-fMRI F-map colorbar is the same over all models. HRF, hemodynamic response function; IRF, impulse response function. If HRF demonstrated a reduced peak and an amplified trough (i.e., blue color-coded HRFs) the *F*-values were assigned with a negative sign. The F-maps were threholded with *p* < 0.0001, i.e., |F| > 8.1. The voxels with |F| > 12.1 met the condition *p*_FWE_ < 0.05. The red color coded HRFs were derived from voxels with positive signed suprathreshold *F*-values. All F-maps are shown following the neurological convention, i.e., left hemisphere on the left side of the axial slice. **(A)** Absolute spectral. **(B)** Relative spectral. **(C)** Absolute spatiospectral. **(D)** Relative spatiospectral.

**Figure 6:**
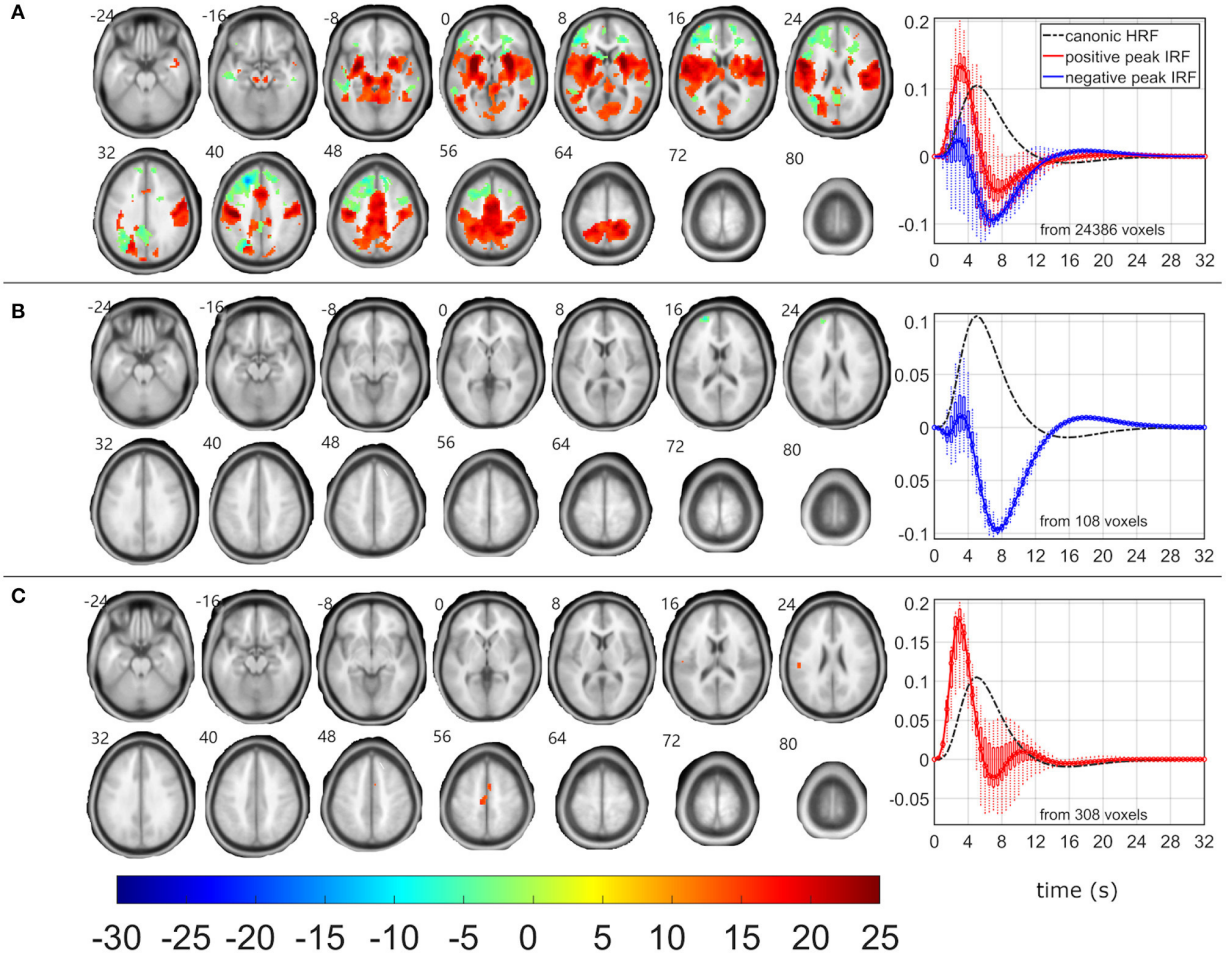
EEG-fMRI F-maps and estimated hemodynamic response functions for all investigated models of the δ_3_ pattern. The EEG-fMRI F-map colorbar is the same over all models. HRF, hemodynamic response function; IRF, impulse response function. If HRF demonstrated a reduced peak and an amplified trough (i.e., blue color-coded HRFs) the *F*-values were assigned with a negative sign. The F-maps were threholded with *p* < 0.0001, i.e., |F| > 8.1. The voxels with |F| > 12.1 met the condition *p*_FWE_ < 0.05. The red color coded HRFs were derived from voxels with positive signed suprathreshold *F*-values. All F-maps are shown following the neurological convention, i.e., left hemisphere on the left side of the axial slice. **(A)** Absolute spectral. **(B)** Relative spectral. **(C)** Absolute spatiospectral. **(D)** Relative spatiospectral.

The RSM, ASSM and RSSM did not demonstrate almost any significant EEG-fMRI associations for δ_2_ and δ_3_ patterns (Figures 5, 6, Table 2). The ASM for δ_2_ and δ_3_ patterns showed similar spatial and HRF observation as for the θ_1_ ASM (Figures 4A, 5A, 6A). The similarity in EEG-fMRI results between δ_2_ and δ_3_ ASMs was expected as both use almost the same spectral filtering properties over all leads (Figure 1).

### Distractor-Related EEG-fMRI Networks

The evidence of a negative relationship between EEG power fluctuations and distractor stimulus was only noticed for the δ_4_ pattern. The δ_4_ EEG-fMRI F-maps might then represent both frequent-related or distractor-related associations (Figure 2).

## Discussion

### Novelty and Neuroimaging Impact

Our results on visual oddball task data represent the systematic objective comparison of spectral (i.e., ASM, RSM) and spatiospectral (i.e., ASSM, RSSM) EEG-fMRI data fusion methods with the variable HRF permitting the variable delay between the immediate EEG following the BOLD signal changes. The automatically quantified effect of the variable HRF in the EEG-fMRI data fusion was remarkable. Both ASM and RSM results gained from the same dataset with fixed canonical HRF were far from reaching *p*_FWE_ < 0.05 in EEG-fMRI statistical parametric maps (18, 43). In contrast, large numbers of voxels in δ_4_ and θ_1_ EEG-fMRI F-maps for all models met the statistical significance condition *p*_FWE_ < 0.05. While accounting for the obtained EEG-fMRI map robustness and due to the involvement of insula, sensory-motor cortex, somatosensory cortex, cuneus, precuneus, basal ganglia, and FPCN (also known as central executive network) in the oddball/P300 tasks (64–73), we recommend using variable HRF and absolute spectral (ASM) or relative spatiospectral model (RSSM) for blind and fully automatic visualization of task-related networks from simultaneous EEG-fMRI data.

The current approach is fully automated, blind, and robust. It overcomes EEG-fMRI fusion *via* event related potential (ERP, e.g., the P300) analysis of amplitudes or latencies (22, 74–76) and is without a need of supplying an input information about stimuli timings. Manpower expertise and efforts are required for the visual identification in the ERP analysis. In the proposed analysis approach, quantitative EEG-fMRI task-related networks are generated automatically. The potential neuroimaging impact of this methodology is in a quantitative measurement of local data-driven determinants of cognitive deficit in patients suffering with epilepsy or other conditions with a cognitive impairment. The specific outcome determinants may be subject/group-specific *F*-value magnitudes or variable HRF amplitudes and latencies. High variance in local HRF latencies has been recently reported in patients with refractory focal epilepsy (40). This observation underlines the importance of variable HRF models for future clinical EEG-fMRI applications.

We demonstrated local EEG-fMRI response functions with the reduced HRF peak and the amplified HRF trough with the most robust location in the center of white matter bundles. These bundles may convey information related to execution and goal-directed tasks (77–79). Although the white matter BOLD signal was considered as a nuisance signal that was usually regressed out from the dataset during the preprocessing (80), several studies have reported the white matter fMRI-BOLD signal and disregarded its previous categorization as a blind spot in the functional imaging (81–85). Our robust white matter EEG-fMRI associations with the reduced HRF peak and the amplified HRF trough corroborate this recent blind spot hypothesis.

Task-related δ_4_ and θ_1_ EEG-fMRI associations might be considered controversial but we are convinced that they represent the bands of major event-related fingerprints in the simultaneously recorded EEG spectra. The postulated event-related fingerprint hypothesis is described in more detail in the following sub-chapter and supported by previous ERP findings.

### Event-Related Fingerprints in EEG Spectra Hypothesis

The oddball paradigm elicits ERPs in EEG recordings (22, 67, 74–76, 86). The averaged ERP can be decomposed at several components of different frequency bands with major contributions of δ oscillations to P300 wave and θ oscillations to P300, P1, and N1 waves (87). Generally, δ and θ oscillations, which underline the P300 wave (88–93), reconfigure and enhance functional connectivity architecture from the baseline resting-state condition to the P300 task condition (73).

Temporal and spectral domains are fully complementary spaces. Therefore, each single event-related change of each oddball stimulus recorded in the EEG temporal domain fingerprints into the spectral domain such as power change in the ERSPat. Then, the task-related networks can be visualized while utilizing power spectral or spatiospectral models in the fully automated approach that is fully blind to the external stimulus timings. Our results demonstrated that the δ_4_ and θ_1_ ERSPats might be key filtering properties, i.e., *g(ω)* in Equation 1 or *g(c,ω)* in Equation 2, which appear to be in correspondence with the spectral properties of the P300 ERP (87–93). Our observation expands beyond the original hypothesis that was simply monitoring changes in the EEG mean root square frequency characterizing a signal roughness increase after stimuli (16, 47). These global EEG spectra changes are tiny and hard to detect in the event-related paradigm design. The ERSPat approach may be beneficial for the assessment of the event-related changes in EEG signal.

We have shortened the term, “event-related (spatio)spectral patterns” to an acronym, “ERSPat” and not “ERSP” to avoid confusion with the ERSP acronym. The “ERSP” acronym is used in the field for the event-related spectral perturbation (94), which are estimated from EEG segments that are apriori time-locked to the stimulus on-sets. Thus, ERSP has a slightly different meaning and interpretation than proposed ERSPats.

### Comparison With Concurrent Brain Network and Early Visual Components

Our results corroborate the previous findings of δ and θ oscillations representing the major operating rhythms in P300 (87–93). Detected β oscillations underlying P300 were likely related to directed attention and cognitive activity (95). The earliest components in ERP with visual sensory input are positive and negative peaks reflecting P1 and N1 potentials and are likely generated in lateral extrastriate occipital cortex, temporo-parietal junction, and fusiform gyrus, respectively (96–98). These components may be linked to visual perceptual processing, especially visuospatial attention. Within the latency window of P1-N1 complex (90–120 ms for P1; and 150–190 ms for N1), the suggested superposition of *α* and θ evoked oscillations (99) was corroborated with the oscillatory activity detected in *α* and θ bands in the frequency decomposition of P300 response (87). In our findings, the activity in the *α*_1_ band approached the borderline of significance for the frequent stimulus and, thus, was not associated with fMRI signals. We believe that the θ activity was likely dominated by the P300-related oscillatory response and the discernibility of the rhythms linked to a minute P1-N1 complex is rather challenging.

The detection and discrimination between stimuli initiate a frontal lobe activity underlying attention-demanding task. The frontal activation interplays with an activation in temporal-parietal areas that promote memory operations. At rest, DMN (hippocampal-cingular-temporal-parietal network) is characterized by high activity. During attention-demanding task DMN activity is suppressed. FPCN is often found to be reciprocally anti-correlated with DMN, which is one of the examples of the functional antagonism (100–103). The involvement (δ_4_ and θ_1_ oscillation related) of the insula, the central node of the salience-detection system, represents an expected “switch” between the DMN and the FPCN (57–60). Observed δ_4_ oscillation related EEG-fMRI associations in the DMN regions are speculative. Predominantly, *α* oscillation related DMN deactivation has been present within the oddball task (18, 19, 60, 73) or resting-state (13). The involvement of the posterior cingulate cortex has been proposed as a potential regulatory modulator of the DMN in the task-negative state (104, 105).

### Comparison With Concurrent Methods Applicable for EEG-fMRI Fusion

Temporal ICA of EEG data can isolate time-locked oscillations, artifacts (e.g., EKG, eye-blinking, etc.) (106) and epileptogenic spikes (if present) (25, 107, 108). The task specific activity represents only a small portion of signal variance, such as high frequency gamma band activity or ERPs. In addition, the present approach isolates EEG responses over large windows (e.g., 1.66 s in the present study), which discards the time-locked activity present with conventional ERP analysis. The time-locked activity preservation, e.g., with group temporal ICA, may also be a promising approach for task data (48).

Yet, considering the hypothesis that event-related changes (i.e., the small portion of signal variance) fingerprints into several distinct spectral patterns (87), then the temporal ICA appears less well-suited at decomposing distinct EEG oscillations (i.e., decompose EEG signal from EEG signal) compared to alternative approaches including (but not limited to) second-order blind identification (109–111), approximate joint diagonalization of cospectra (112, 113), and spectral ICA (114, 115).

The presented GLM EEG-fMRI fusion approach with variable HRF significantly increased robustness of obtained results for all investigated models compared to the same dataset observations proposed with ASM/RSM with the fixed HRF (18). The dominating fixed HRF GLM approach (5–9, 11–18, 21–26, 28–33, 43) may be revisited to gain an increased robustness of the results enabling variable HRF timings or utilizing an EEG-fMRI deconvolution approach (36–38). Similarly, this approach can be adopted for epileptogenic focus localization (25, 107, 108) due to the fact that the deconvolution approach demonstrated that delay timings between ictal EEG-fMRI associations does not fit to canonical HRF timings and often the local BOLD signal even precedes the EEG spike (41, 42).

The voxel-wise GLM approach is not the only data processing strategy to compare and fuse the EEG and fMRI data. The spatial ICA can rotate fMRI data into a space of spatially independent large scale brain networks (116, 117) with a representative component-specific time-course, which can be associated with simultaneously acquired EEG signal transformed into a comparable signal form (i.e., undersampled to fMRI timings, power fluctuations, spike timings/delays, etc.) (27, 30). Utilizing sliding windows over both EEG and fMRI-BOLD time-courses can be applied in the estimation of dynamic functional connectivity associations (34). A graph matrix between EEG and fMRI measures may be build *via* correlation measures or other similarity criteria (33). EEG-fMRI mixing parameters can be estimated through joint analysis approaches such as the joint-ICA (32, 118–120).

Over various existing EEG-fMRI data fusion strategies, we have demonstrated a fully automated GLM approach for robust lateralized task-related network visualization from local fMRI-BOLD signals and spectrally distinct task-related EEG patterns. In our approach, the variable HRF significantly improved the final robustness as the modeled EEG-fMRI peak delay did not overlap with the canonical HRF peak delay for the most task-related spectral patterns. Therefore, we propose this method for future clinical research applications in patients with neurocognitive deficits. These outcomes may also derive objective disease specific markers through local F-statistics or HRF changes that can be potentially used for the diagnostics, disease severity and treatment effect evaluations.

### Limitations and Future Work

From the set of tested models, the ASM and RSSM were the most promising and robust for the blind visualization of task-related networks derived from simultaneous EEG-fMRI data. Several similar spectral models (16, 17, 47) considered Equation 10 instead of using Equation 1 for the EEG-fMRI fusion formulation, sometimes not-using the function *g(*ω*)*. Comparison to the Equation 10 spectral model was not addressed here. All investigated EEG patterns were narrow band-pass filters and the effect of the non-linear *ω*^2^ modulation were considered minimal. The ASM and RSSM have also not been directly compared to the concurrent recent data-driven EEG spectra decomposition approaches such as parallel factor analysis (PARAFAC) (20, 30, 121), coupled matrix-tensor factorization (40, 122), coupled tensor-tensor decomposition (123), or source-space ICA (124, 125). A full comparison to the most recent EEG-spectra fusion strategies should be addressed in the future research. Yet the variability in HRF yielded more significant effects with all four models than in our previous work (18).

(10) $$b\propto \left(\int \omega^{2}g(\omega )p(\omega )\;d\omega \right)*h$$

The robust task-related EEG-fMRI F-maps might be an effect of the active push-button response to the target stimuli. The maps' robustness may decrease using target count or passive responses (126) but such investigation was beyond the investigation of the current dataset and the study scope. The F-maps can be interpreted as data-driven functional connectivity maps (127, 128) where the EEG pattern's fluctuations emphasize the common relationships with local variable HRFs. The effective connectivity (127, 128) has not been quantified and evaluated here. Dynamic causal modeling (DCM), mostly between pre-selected regions of interest (ROIs), belongs to one of the most actively developed procedures quantifying the effective connectivity in fMRI or EEG data (129–132). Recently, the Bayesian fusion and multimodal EEG-fMRI DCM substantially improved the best effective connectivity model evidence (35). Future test-retest at ROIs of robust visual oddball data can provide important evidence of the DCM applicability.

Although the ASM provided the most robust spatial results, there are two weaknesses in the current ASM approach: (i) All ASM filtering properties were derived as an spectral average of the ASSM and RSSM patterns. Therefore, there would not be any optimized spectral filtering property without prior EEG ASSM and RSSM data-driven estimation *via* the spatiospectral ICA. Still, it appears that simple band-pass filters might be a sufficient approximation for the most promising δ_4_ and θ_1_ patterns. (ii) Thresholded ASM spatial F-maps appeared very similar over different EEG patterns. That might be an effect of a reported broadband component in the absolute EEG spectra resulting in lower spatial specificity of the ASM. On the other hand, high spatial specificity might be obtained with stricter statistical threshold, e.g., *p*_FWE_ < 0.01 or even more stricter, which, however, would not be applicable for other fusion models.

Several EEG patterns demonstrated similar spatial EEG-fMRI F-maps while variable HRF was modeled in each voxel of each EEG band. It was not optimized and tested whether a weighted (*w*_*i*_∈ <-1,1>) mixture of *N* EEG patterns (***u***_*i*_) would increase a linear dependence (i.e., |t|-values) between the final EEG pattern and the stimulus vector (***x***), see Equation 11. Then, a general linear mixture EEG-fMRI fusion model with one global but still variable HRF would appear as Equation 12. Similar model analogy has been tested on hand-grip task data and compared to a single band EEG pattern with variable HRF (39). The results demonstrated more significant and specific blind task-related network visualizations for single δ and θ bands with band-specific variable HRFs. It is basically the same result presented here on the visual oddball dataset. Considering a linear mixture of EEG patterns with pattern specific HRFs maximizing relationships to the task, the Equation 12 would change to Equation 13. The optimization of the right side of the Equation 13 regarding the maximized linear dependence to the stimulus vector is challenging. Such EEG-fMRI fusion model alterations (i.e., Equations 12, 13) require a further investigation and testing on visual oddball data within the future research.

(11) $$w=\text{arg max }t\left(\sum_{i=1}^{N}w_{i}u_{i},x\right)$$

(12) $$\begin{array}{l} \text{b}\propto \overset{N}{\underset{i=1}{\sum }}w_{i}{\text{u}}_{i}*\text{h} \end{array}$$

(13) $$\begin{array}{l} \text{b}\propto \overset{N}{\underset{i=1}{\sum }}w_{i}{\text{u}}_{i}*{\text{h}}_{i} \end{array}$$

Although temporal and spectral domains are considered fully complementary, the phase information of the spectral domain in all implemented EEG-fMRI fusion models was omitted here. It is a common procedure in the field to only utilize the magnitude information (5, 7, 10, 16, 17, 26). We suggest that the phase coupling effect should not be neglected in the future research. The phase coupling can separate different sources of similar magnitude/power profiles and it has already been successfully implemented in several EEG spectra blind source separation techniques (112–114, 133–136) including the used spatiospectral ICA (37, 137).

Our proposed experiments and the comparison of the fully automated and blind methodological approaches were tested in healthy subjects. The clinical impact needs to be determined in future studies of the electrical neural and neurovascular associations measured by simultaneous EEG-fMRI. The P300 ERP has been extensively investigated in studies of cognition in healthy individuals and in a wide range of neurological or psychiatric disorders. The lower amplitude and longer latency are measures indicative of slowing of general cognitive ability due to the disease condition (100, 138). We propose that local F-map or HRF changes may discern patients with a specific cognitive dysfunction and improve spatial specificity of dysfunction focus as a benefit of the fMRI resolution in future applications.

## Data Availability Statement

The data analyzed in this study is subject to the following licenses/restrictions: Data are property of the 1st Department of Neurology, St. Anne's University Hospital Brno, Czech Republic. Data can be made available upon request by email. Requests to access these datasets should be directed to Milan Brázdil, milan.brazdil@fnusa.cz.

## Ethics Statement

The studies involving human participants were reviewed and approved by Masaryk University Ethics Committee, Brno, Czech Republic. The patients/participants provided their written informed consent to participate in this study.

## Author Contributions

RL implemented ASSM and RSSM and wrote the first draft of the manuscript. ZW implemented ASM and RSM, compared all models, designed manuscript graphics and tables, and revised the manuscript. DB supervised the blind source separation of EEG spectra and revised the manuscript. MB led the EEG-fMRI data acquisition and preprocessing, approved the neurophysiological validity of the results, and revised the manuscript. JJ supervised the EEG-fMRI data fusion method implementation, confirmed signal processing validity of implemented methods, and revised the manuscript. IN supervised the current model comparison research, approved the neurophysiological validity of the results, and significantly revised the first draft of the manuscript. All authors contributed to the article and approved the submitted version.

## Conflict of Interest

The authors declare that the research was conducted in the absence of any commercial or financial relationships that could be construed as a potential conflict of interest.
